# Impact of sanitary conditions and dietary amino acids on behaviour and brain monoamine levels in piglets

**DOI:** 10.1016/j.bbih.2025.101076

**Published:** 2025-08-05

**Authors:** Ilaria Minussi, Walter J.J. Gerrits, Alfons J.M. Jansman, George Troupakis, Marlies Diepeveen-de Bruin, Yannick Vermeiren, J. Elizabeth Bolhuis

**Affiliations:** a Adaptation Physiology Group, Department of Animal Sciences, Wageningen University & Research, P.O. Box 338, 6700 AH, Wageningen, the Netherlands; b Animal Nutrition Group, Department of Animal Sciences, Wageningen University & Research, P.O. Box 338, 6700 AH, Wageningen, the Netherlands; c Wageningen Livestock Research, P.O. Box 338, 6700 AH, Wageningen, the Netherlands; d Division of Human Nutrition and Health, Chair Group of Nutritional Biology, Wageningen University & Research, P.O. Box 17, 6700 AA, Wageningen, the Netherlands

**Keywords:** Pig, Amino acids, Choice-feeding, Hygienic conditions, Animal behaviour, Salivary cortisol, Serotonin, Brain neurotransmitters, Tryptophan, Inflammation

## Abstract

**Background:**

Immune activation due to poor hygienic conditions may affect behaviour, as the immune system and brain are linked through several mechanisms. This putative effect may partly result from increased amino acid (AA) metabolism, affecting brain monoamine levels. As pigs can select a diet that meets their AA requirements, offering them the choice between an AA deficient and AA enriched diet may influence the effects of sanitary conditions on brain neurochemistry and behaviour.

**Methods:**

We investigated the behavioural and neurochemical effects of high (HSC) vs. low (LSC) sanitary conditions on pigs with or without a choice to select between two diets differing in AA concentration in a 2x2 factorial experiment. Female pair-housed piglets (n = 48) were kept under HSC or LSC and were offered either a diet deficient in eight indispensable AA or the choice between the deficient diet and a diet enriched with these AA for 19 days. Behaviour, salivary cortisol, blood tryptophan (Trp) and serotonin (5-HT), and hippocampal/prefrontal concentrations of Trp, related monoamines and their metabolites were analysed.

**Results:**

LSC had lower platelet serotonin and plasma Trp than HSC piglets. Hippocampal and prefrontal cortex concentrations of Trp, kynurenine (KYN), kynurenic acid, epinephrine, norepinephrine, 5-HT, dopamine (DA), 5-hydroxyindoleacetic acid and homovanillic acid (HVA) were lower for LSC than HSC piglets. LSC piglets had lower KYN/Trp ratios in the hippocampus and, if without dietary choice, in the prefrontal cortex. LSC piglets showed less play behaviours. Piglets without a dietary choice had lower plasma Trp, higher hippocampal HVA/DA turnover, and spent more time on damaging behaviours. No effects on salivary cortisol were found.

**Conclusions:**

A decline in health status induced by poor hygienic conditions reduced platelet 5-HT, blood and brain Trp, as well as brain neurotransmitter concentrations, and negatively influenced behaviour of pigs. There was no impact of offering a choice of an AA-enriched or AA-deficient diet on these effects of sanitary conditions, possibly due to low intake of the AA-enriched diet.

## Highlights

• We studied choice-feeding of an amino acid supplemented vs deficient diet in piglets.
• Piglets were kept under low (LSC) or high sanitary conditions (HSC) after weaning.
• Blood and brain tryptophan, and brain monoamine levels were lower in LSC than HSC.
• LSC reduced play behaviours compared to HSC.
• Damaging behaviours were lower in piglets with dietary choice than in piglets without.

## 1 Introduction

Low sanitary conditions due to poor hygiene measures modify the metabolism of amino acids (AA) not only by increasing their oxidation, but also by redirecting them from body protein retention towards inflammatory and immunological processes (Le Floc'h et al., 2004; Melchior et al., 2004; Le Floc'h et al., 2009; Kampman-van de Hoek et al., 2016; van der Meer et al., 2016; Alves da Cunha Valini et al., 2023). The immunological changes resulting from such conditions therefore alter the requirements of AA and the optimum ratios between different AA (Le Floc'h et al., 2004). As AA are involved in many physiological functions, insufficient or imbalanced dietary intake of AA has been shown not only to impair body protein retention, but also to increase aggressive and stress-related behaviours (van der Meer et al., 2016; van der Meer et al., 2017; Sato et al., 2020; Alves da Cunha Valini et al., 2023). Some AA are indispensable (IAA) and must be obtained through the diet, whereas dispensable AA (DAA) can be synthesised endogenously from other AA, indicating that only dietary intake can meet the increased demand for IAA during immune activation.

Health and behaviour seem causally linked via a series of metabolic and neurochemical mechanisms that involve IAA (Nordgreen et al., 2020). Firstly, cytokines can alter brain neurotransmission (Dantzer et al., 2008; Lasselin and Capuron, 2014). Pro-inflammatory cytokines, for example, shift the catabolic fate of the IAA tryptophan (Trp) toward the inflammatory-prone kynurenine (KYN) pathway rather than to the hydroxylation pathway. This shift leads to increased production of KYN due to elevated activity of indoleamine 2,3 deoxygenase 1 (IDO) to support the immune response. Accordingly, as Trp is the precursor of serotonin (5-hydroxytryptamine; 5-HT), this will lead to lower 5-HT levels in the brain (Lestage et al., 2002; O'Connor et al., 2009; Walker et al., 2013). This may consequently influence behaviour and affective state, given that brain serotonergic neurotransmission is involved in their regulation, as demonstrated in humans, rats, and pigs (Carrasco and Van de Kar, 2003; Carr and Lucki, 2010; Poletto et al., 2011; Ursinus et al., 2014). By contrast, catecholamines such as dopamine and norepinephrine derive from tyrosine, a DAA that can be endogenously synthesised from phenylalanine. Also brain concentrations of other neurotransmitters may be affected by poor health. Recent studies in pigs and mice observed that intraperitoneal or intravenous injection with lipopolysaccharide (LPS) to trigger a sickness response reduced the noradrenergic, dopaminergic, and serotonergic neurotransmitter concentrations in different brain areas (frontal cortex, hypothalamus, hippocampus) (Zhu et al., 2015; Nordgreen et al., 2018; Veit et al., 2021). In these and other studies, LPS challenged animals with lower levels of neurotransmitters showed depression- and sickness-like symptoms, and increased aggression and other damaging behaviours (Valros et al., 2015; Zhu et al., 2015; Munsterhjelm et al., 2019; Veit et al., 2021). A second reason for a causal link between health and behaviour that involves protein and AA metabolism is the impaired food intake and nitrogen digestibility resulting from poor health (Hart, 1988; Le Floc'h et al., 2004; Noorman et al., 2023), increasing the likelihood of immune-stimulated individuals developing nutritional deficiencies. Such nutritional deficiencies may, in turn, induce changes in behaviour. In pigs fed an IAA deficient diet, such behavioural changes encompass increased activity levels and foraging behaviours like rooting, likely reflecting a search for alternative feed items under natural conditions. In commercial pig production, where substrates to root in are scarce and alternative feed sources absent, foraging activities can be redirected towards pen mates in the form of tail and ear biting, thus increasing the risk of outbreaks of these damaging behaviours (van der Meer et al., 2016, 2017; Nordgreen et al., 2020; Boyle et al., 2022; Minussi et al., 2023). Dietary supplementation of IAA has been shown to mitigate the negative effects of an acute or chronic activation of the immune system on growth performance and to decrease the occurrence of aggression and other damaging behaviours in pigs, especially under low sanitary conditions (van der Meer et al., 2016; van der Meer et al., 2017; Alves da Cunha Valini et al., 2023). In addition, supplementation of specific IAA, such as Trp, has been shown to reduce stress and aggressive responses in pigs (Li et al., 2006; Martínez-Trejo et al., 2009; Poletto et al., 2014). This indicates the potential of exploring nutritional interventions to prevent undesirable effects of low sanitary conditions on behaviour and mood in pigs.

Pigs can select a diet that meets their AA requirements over an AA-deficient diet when given the choice under non-challenged conditions (Ettle and Roth, 2004, 2005, 2009; Roth et al., 2006; Suarez et al., 2012; Minussi et al., 2024). However, it is unknown whether offering pigs the choice to select between an AA-deficient diet and an AA-enriched diet, potentially compensating for the increased usage of AA due to immune stimulation, will affect the impact of sanitary conditions on brain monoamines and behaviour.

Hence, the objective of the present study was to investigate the behavioural and neurochemical effects in the brain of piglets kept under different sanitary conditions (high vs. low) and offered either a diet deficient in AA, or the choice between this deficient diet and an AA-enriched diet. Piglets’ behaviour, salivary cortisol, blood Trp and serotonin, and concentrations of Trp, monoamines and their metabolites in the hippocampus and prefrontal cortex were analysed. Apart from damaging behaviour, play behaviour was recorded as this behaviour is seen as an indicator of a positive affective state (Boissy et al., 2007; Stracke et al., 2017). It was hypothesized that piglets kept under high sanitary conditions would display increased play behaviour, decreased aggressive and damaging behaviours, and show higher noradrenergic, serotonergic, and dopaminergic neurotransmitter levels compared to piglets kept under low sanitary conditions. In addition, it was hypothesized that providing piglets with a dietary choice would have beneficial effects on behaviour and increase brain monoamine levels, particularly in pigs under low sanitary conditions.

## 2 Materials and methods

The study was authorized by the Dutch Council on Animal Experiments (CCD), and experimental procedures were approved by the Animal Welfare Body of Wageningen University & Research (AVD10400202216386). The experiment was conducted at Wageningen University and Research between September–November 2023. Piglets were exposed to one of the four experimental treatments for a period of 19 days. In a 2 × 2 factorial design, pairs of piglets were allocated to either high sanitary conditions (HSC) or low sanitary conditions (LSC) and were offered either a diet deficient in indispensable AA (IAA) (No dietary choice) or were given the choice between the IAA-deficient and an IAA-enriched diet (Dietary choice). Further details are provided below. Low sanitary conditions were demonstrated to decrease feed intake and growth, and cause a low-grade elevation of immune activity of the piglets without signs of clinical illness (Minussi et al., 2025).

### 2.1 Animals, housing, and management

The experiment was carried out using two batches of animals from a larger experiment (Minussi et al., 2025). A total of 48 female piglets (Topigs Norsvin, TN70 x Tempo), offspring of 12 sows, were used over an experimental period of 19 days. Piglets were weaned at four weeks of age (mean ± SD: 8.35 ± 0.79 kg body weight (BW)) on a commercial farm. On the weaning day, four female piglets closest to their litter's mean BW were selected from each litter. After selection, piglets were transported to the experimental facilities and housed in one of four climate respiration chambers containing three pens each. Piglets were housed in pairs of non-littermates. The four piglets from each litter were distributed over the four treatments in such a way that between-treatment BW differences and BW variation between pairs among treatments was minimized. Initial BW did not differ per treatment (8.09 ± 0.77 kg). Two chambers were dedicated to each sanitary status treatment and within each chamber, two pens were assigned to the Dietary choice treatment and one to the No dietary choice treatment, leading to four replicates for the HSC-No dietary choice and LSC-No dietary choice treatments, and eight replicates of the HSC-Dietary choice and LSC-Dietary choice treatments, as more variation was expected within the Dietary choice groups which could select their own diet. Pens (0.88 × 2.88 m) had a partially slatted floor and contained a nipple drinker and a feeder. Each feeder had two separate feeding spaces (25 × 25 cm). Pens contained a plastic toy (PorkyPlay, Ketchum Manufacturing, Lake Luzerne, NY, USA) and a metal chain (90 cm) intended as enrichment materials. Piglets had *ad libitum* access to the experimental diets and water. Lights were on between 07.00 and 19.00 h. Room temperature gradually decreased from 28.0 °C to 25.3 °C and relative humidity decreased from 65.0 % to 62.3 % from transport to the end of the experimental period. Ventilation was set at 18 m^3^/h. The experimental period started the day after arrival on the experimental farm, and it is referred to as d 0.

### 2.2 Sanitary conditions

The protocol used to impose sanitary conditions was adapted from a previous study (Noorman et al., 2023). The LSC treatments were obtained by spreading twice a week 1.5 kg of faeces in each pen. The faeces were collected from pens of weaned piglets of four commercial farms. Faeces from each farm were collected separately from multiple rooms, and faeces from each room were homogenized and sampled for analysis of the presence of pathogens. After the sampling procedure, faeces from each department were mixed with a 0.9 % NaCl solution (2:1 w/w) and glycerol (12 % w/w) before storage at −20 °C. The samples were analysed for *Clostridium perfringens* toxin CPA, CPB, CPB2, *Escherichia coli* virulence factor F4, F42, F5, F6, and Rotavirus A with q-PCR (Dutch National Health Service for Animals Royal GD, Deventer, The Netherlands). Faecal subsamples with two or more of the analysed pathogens at critical level (4 out of 13 samples) were excluded to minimize the risk of introducing diseases (see Supplementary Table S1 for results of the analysis). The remaining faecal samples were pooled into one large batch, homogenized, and divided over buckets containing 1.5 kg of diluted pooled faeces each, and stored at −20 °C. The buckets containing pooled faeces were thawed overnight at room temperature for each timepoint before being spread into the LSC pens (d 0, 4, 7, 11, 14, and 18). One bucket per timepoint was used for each LSC pen. In the HSC treatments, manure was removed daily from the pens. To maintain the imposed sanitary condition within a room, the rooms of HSC and LSC had their own entrance, manure pit, and ventilation system (Heetkamp et al., 2015). Prior to the arrival of the piglets, chambers of both treatments were cleaned with high-pressure washing, but only the HSC chambers were treated with disinfectants (Desbest400, Veugen Technology B.V., Nederweert, The Netherlands; Virocid, CID LINES N.V., Ieper, Belgium). In addition, a strict hygiene protocol was applied when entering the HSC rooms, which included showering, change of clothes, and use of a hairnet and face mask. These procedures were not applied to the LSC chambers.

### 2.3 Diets

From experimental d 0, piglets were assigned to one of the two dietary treatments. In the No dietary choice treatment piglets were fed a diet deficient in IAA (LP^-^), while in the Dietary choice treatment piglets could choose between the LP^-^ diet and a diet supplemented with IAA above requirements for maximal body weight gain (LP^+^). The LP^-^ diet was formulated to be deficient in Lys, Met, Thr, Trp, Val, Leu, Ile, His by 20 % compared to requirements for maximal body weight gain (Central Bureau for Livestock Feeding, 2020). The LP^+^ diet was formulated to have Lys, Met, Thr, Trp, Val, Leu, Ile, His at 20 % above the requirement values for maximal body weight gain (Central Bureau for Livestock Feeding, 2020). To obtain the LP^+^ diet, the LP^-^ diet was supplemented with free L-Lys, DL-Met, L-Thr, L-Trp, L-Val, L-Leu, L-Ile, and L-His at the expense of maize starch. Despite being an IAA, L-Phe was not supplemented due to its limited use in commercial pig diets. The diets were isocaloric on a NE basis. Diets were given in pelleted form. The ingredient and nutrient composition of the diets are shown in Supplementary Table S2. For more details on the diet formulation, see our previous paper (Minussi et al., 2025).

In the Dietary choice treatments, diets were formulated so that piglets could meet their AA requirements for maximal body weight gain by ingesting the LP^+^ and LP^-^ diets in a certain ratio according to their free choice. In the No dietary choice treatments, the diet was divided over the two feeding spaces, while for the Dietary choice treatment each of the two diets provided was given in one of the two feeding spaces. The position of the two diets of the Dietary choice treatment within the feeders was changed on d 4 and 11 to avoid an effect of feeding place preference on diet choice.

## 3 Measurements

### 3.1 Behavioural observations

Behavioural observations were performed on d 16. Individual piglets within a pen were identified by coloured spray mark on their back. Throughout the experiment, a camera attached to the ceiling of each chamber recorded the piglets continuously. Frequencies and durations of the behaviours described in the ethogram in Table 1 were recorded with continuous observations of videos during the light period from 07.00 to 19.00 h. In case a piglets paused performing a behaviour for more than 5 s, a new occurrence started. Recordings were analysed with The Observer XT 16 software (Noldus Information Technology B.V., Wageningen, The Netherlands). While a person was inside a pen for routine controls, any behaviour performed by the piglets was excluded from the analysis. The behavioural observations were all performed by the same person, who was blind to dietary treatments but not to the sanitary conditions.

**Table 1:** Ethogram of the recorded piglet behaviours.

Item	Description
Object play	Energetic manipulation (shaking, tugging, throwing) of object with the mouth or snout; Forward movement while carrying object which protrudes from mouth (Newberry et al., 1988; Bolhuis et al., 2005)
Individual locomotor play	
Gambolling and scamper	Running across the pen, occasionally bouncing into pen mate that does not join (gambolling); two or more forward hops in quick succession (scamper) (Martin et al., 2015)
Hop	Jump on the spot, while facing one direction (Newberry et al., 1988)
Pivoting	Turning around the body axis, often accompanied by jumping (Martin et al., 2015)
Rolling	Lying on back and moving from side to side (Bolhuis et al., 2005)
Toss head	Energetic movements of the head and neck in succession (Newberry et al., 1988; Martin et al., 2015)
Social locomotor play	
Push	Mildly pushing pen mate with head, neck, or shoulders (Martin et al., 2015)
Nudge	Gently nudging pen mate's body (Martin et al., 2015)
Running together	Following the ‘gambolling and scamper‘ behaviour of pen mate
Play fight behaviour	Similar to fighting behaviour (see below), but milder and without biting; often with simultaneous manifestation of at least one element associated with individual or social locomotor play (Cordoni et al., 2022)
Damaging behaviour	
Tail biting	Oral manipulation of the tail of pen mate, including sucking, nibbling, chewing, and biting (Bolhuis et al., 2005)
Ear biting	Oral manipulation of the ear of pen mate, including nibbling, chewing, and biting (Bolhuis et al., 2005)
Other damaging behaviour	Oral manipulation of another body part of pen mate, including nibbling, chewing, and biting (Bolhuis et al., 2005)
Aggressive behaviour	
Fighting	Mutual pushing, ramming, or lifting pen mate, with or without biting (Bolhuis et al., 2005; Cordoni et al., 2022)
Attack	One-sided ramming or pushing pen mate with the head, and/or biting (Bolhuis et al., 2005; Chaloupková et al., 2007)

### 3.2 Saliva collection and analysis

At d 2, 11, and 18 of the experiment saliva samples were collected to measure cortisol concentrations. Piglets were allowed to chew for around 2 min on polypropylene swabs (Salivettes®, Sarstedt Inc 51.1534.500) held by a clamp forceps. Saliva samples were collected between 8h00 and 8h30 by two teams, each consisting of two individuals, simultaneously operating under the two different sanitary conditions. Piglets were previously habituated to the procedure at the start of the experiment. The number of saliva samples collected for each treatment on d 2, 11, and 18 was respectively: HSC-No dietary choice = 8, 8, 8; HSC-Dietary choice = 16, 14, 13; LSC-No dietary choice = 8, 8, 8; LSC-Dietary choice = 13, 16, 15. Salivary cortisol was measured using the cortisol kit (Enzyme immunoassay for the quantitative determination of free cortisol in human saliva, ref RE52611) from IBL International GmbH (Hamburg, Germany).

### 3.3 Blood collection

A blood sample was taken from all piglets on experimental d 18 from the jugular vein. Samples were collected in a 1 mL and 9 mL ethylenediaminetetraacetic acid (EDTA) tubes (Vacuette; Greiner Bio-One, Kremsmünster, Austria) and used to determine platelet 5-HT level, whole blood 5-HT, and free Trp in plasma. The number of blood samples per treatment ranged as follows depending on the analysis: HSC-No dietary choice = 6–8; HSC-Dietary choice = 10–14; LSC-No dietary choice = 6–8; LSC-Dietary choice = 12–14.

### 3.4 Serotonin measures

*Collection of platelets and platelet count.* Blood samples collected in EDTA tubes were immediately stored on ice and transported to the lab. The 9 mL tubes were centrifuged at 160×*g* for 10 min at room temperature without braking. Approximately 5 mL of plasma was removed and centrifuged at 1300×*g* for 15 min at room temperature. Using this soft spin, the platelet rich plasma (PRP) formed a layer between the platelet poor plasma and red blood cells, and approximately 1.5 mL of PRP was yielded. The remaining (platelet poor) plasma was stored at -80 °C for Trp analysis (see below). From the approximate 1.5 mL of obtained PRP, 0.5 mL was kept for platelet count and 1 mL was kept for 5-HT analysis. The 0.5 mL PRP samples were stored for 24 h at room temperature before platelet count, and the same held for the 1 mL EDTA whole blood sample that was taken. Whole blood and PRP platelet counts were determined with a cell counter (ADVIA® 2120i Hematology System with Autoslide, Siemens Healthineers AG, Forchheim, Germany).

Tubes containing 1 mL of PRP were centrifuged for 13,000×*g* for 15 min at 20 °C to obtain platelet pellets. The pellets were washed with 1 mL of 0.9 % NaCl solution and centrifuged at 13,000×*g* for 5 min at room temperature. After removal of the supernatant, platelet pellets were stored at -80 °C until neurochemical analysis.

*Serotonin analysis,* Platelet serotonin was determined using a fluorimetric assay as previously described (Ursinus et al., 2014). Briefly, after thawing the platelet pellets, perchloric acid (HPClO_4_) was added (500 μl). Supernatant was retrieved by centrifuging the samples (13,000 g × 5 min). The supernatants (100 μl) were added in duplicate to a cysteine (1 mL) and ortho-phthalaldehyde (1 mL) solution and incubated in a water bath (80 °C × 20 min). After cooling down on ice (to 20 °C) fluorescence was measured at 360 nm excitation and 475 nm emission with a SpectraMax iD3 (Molecular Devices, San Jose, CA, USA). The 5-HT level of the supernatants was determined by using a linear standard curve with known quantities of 5-HT. Serotonin in platelets represents >95 % of 5-HT found in blood (Da Prada and Picotti, 1979; Celada et al., 1994; Pussard et al., 1996). Therefore, we multiplied platelet 5-HT level by the number of platelets counted in whole blood (10^9^ cells/l) to obtain whole blood 5-HT. Platelet 5-HT level was expressed in nmol/10^9^ platelets and whole blood 5-HT in nmol/mL.

### 3.5 Tryptophan analysis in plasma

Trp in plasma was determined by liquid chromatography mass spectrometry. Approximatively 50 μL of plasma was used for the analysis. Proteins were precipitated by diluting the plasma 1:4 with methanol containing 0.1 % (v/v) of formic acid, mixed and left on ice for 30 min. After centrifugation at 20000 RCF for 10 min, the clear supernatant was diluted 20 times in phosphate buffered saline and d5-Trp was added to a final concentration of 0.5 μmol/L as internal standard. The concentration of Trp was measured using liquid chromatography tandem mass spectrometry (LCMS) on a Waters Acquity Ultra Performance LC system coupled to an Quattro Premier XE triple quadrupole LCMS (Waters Corporation, Milford, MA, USA) using an Accucore PFP column, 2.6 μm, 2.1 mm × 100 mm with matching guard column (Thermo, Waltham, MA, USA). Separation was performed using a gradient, mobile phase A consisted of 0.1 % formic acid in ultra-pure water, mobile phase B was 0.1 % formic acid in acetonitrile, the flow was set to 0.4 mL/min, 5 %B for 2.5 min after which B was ramped to 30 % over 2 min. Finally, the column was washed for 2 min using 80 %B and equilibrated to initial conditions for 3 min. Mass spectrometry was performed using electrospray ionization operated in positive ion mode. The following source parameters were used; capillary voltage 3 kV; source temperature 120 °C, desolvation gas temperature 450 °C at 850 l/h (N2); cone gas at 50 l/h (N2). Nitrogen (99.9 % purity) and argon (99.9999 % purity) were used as cone and collision gases, respectively. Measurements were performed as multiple reaction monitoring (MRM), optimized for Trp (Supplementary Table S3). Interscan delay was set to 50 ms. Data acquisition was performed using the MassLynx V4.1 software (Waters, Waltham, MA, USA).

### 3.6 Brain tissue collection and analysis

On experimental d 18, piglets were euthanized for brain tissue collection. The prefrontal cortex and hippocampus were selected for collection because of their role in the control of behaviour and mood regulation (Damasio, 1995), and of cognition and memory, respectively (McClelland et al., 1995). Order of euthanisation during the day was balanced based upon treatment, and pairs of piglets from one pen were taken together to minimize stress responses. Prior to dissection, piglets were sedated with 10 mg/kg BW ketamine (100 mg/mL, Alfasan, Woerden, The Netherlands) and 0.75 mg/kg BW Midazolam (5 mg/mL, Actavis, Parsippany-Try Hills, NJ, US) through an intramuscular injection. After sedation, piglets were euthanized by an intracardiac injection of 100 mg/kg BW of sodium pentobarbital (Euthasol 40 %, AST Farma BV, The Netherlands). Brains were removed from the skulls and dissected within 20 min postmortem. The prefrontal cortex and hippocampus areas were dissected from the right brain hemisphere based on the landmarks of a pig's brain atlas (Félix et al., 1999). After collection, samples were immediately snap frozen in liquid N_2_, kept in dry ice, and stored at -80 °C until further processing. The number of brain samples per treatment in both the hippocampus and prefrontal cortex was: HSC-No dietary choice = 8; HSC-Dietary choice = 14; LSC-No dietary choice = 8; LSC-Dietary choice = 16.

### 3.7 Neurochemical analysis of brain neurotransmitters and metabolite levels

Trp, kynurenine (KYN), kynurenic acid (KA), serotonin (5-HT), 5-hydroxyindoleacetic acid (5-HIAA), dopamine (DA), epinephrine (EPI), norepinephrine (NE), and homovanillic acid (HVA) concentrations were measured in frozen hippocampus and prefrontal cortex of the right brain hemisphere by means of liquid chromatography-tandem mass spectrometry (LC-MS/MS).

The ultra-performance liquid chromatography (UPLC) system employed was a Waters ACQUITY H-class PLUS system, coupled with an ACQUITY UPLC HSS T3 column (150 mm × 2.1 mm x 1.8 μm). The mass spectrometer (MS/MS) was an Xevo TQ-S micro triple quadrupole MS equipped with an electrospray ionization (ESI) source (Waters™). Data acquisition and analysis were performed using Mass Lynx V4.2 SCN1001 software. Data was expressed in ng neurotransmitter or metabolite per g of wet weighed tissue.

The optimized method utilized deionized, analytical grade water with 0.6 % formic acid (FA) (Eluent A) and methanol with 0.6 % FA (Eluent B). The gradient conditions were: 0 min: 100 % A/0 % B, 0.5 min: 100 % A/0 % B, 3.5 min: 65 % A/35 % B, 4 min: 45 % A/55 % B, 10 min 0 % A/100 % B, 12 min 0 % A/100 % B, 13 min: 100 % A/0 % B, and 15 min: 100 % A/0 % B. Total runtime was 15 min for each injection. Flow rate was maintained at 0.2 mL/min, the column temperature at 55 °C, the autosampler at 5 °C and 5 μL was injected per run. For MS settings, the capillary voltage was set to 3.5 kV, desolvation temperature to 600 °C, desolvation gas flow to 900 L/h, and cone gas flow to 100 L/h for positive mode. All analytes were measured in positive ion mode.

Standards (analytical grade) were DA hydrochloride, 5-HT hydrochloride, (−)-EPI, HVA, KA (≥98 %), KYN (≥98 %), (−)-NE (≥98 %), L-Trp (≥98 %) and 5-HIAA (≥98 %) (Sigma-Aldrich). Solvents used included ULC/MS-grade water, -methanol, -acetonitrile, absolute ethanol, and formic acid (≥99 %) (Biosolve Chemicals, Netherlands). A standard stock solution was made containing all standards. Internal standards were DA-d4 hydrochloride, 5-HT-d4 hydrochloride, NE-D6 (stable isotope-labeled APIs) (LGC Standards Ltd., UK), KA-d5 (Sanbio, Netherlands), and 2H5-L-Trp (≥98 %) (ALSA CHIM, France). From the standard stock solution, a series of 8 standards was prepared (concentrations of 0.40, 1.20, 3.70, 11.11, 33.33, 100.00, 300.00, and 900.00 ng/mL), with each standard containing 40 ng/mL of IS solution.

Specific MRM transitions, collision energies, and cone voltages were optimized for each neurotransmitter and internal standard as shown in Table 2.

**Table 2:** Multiple reaction monitoring (MRM) transitions, collision energies, cone voltages, and dwell time optimization used for each neurotransmitter and internal standard.

Compound	MRM transition (m/z)	Collision energy (V)	Cone voltage (V)	Dwell time (s)
DA	154.11 → 137.06	10	16	0.025
EPI	184.12 → 166.07	8	14	0.025
HVA	183.15 → 137.04	8	28	0.019
KA	190.07 → 143.98	16	18	0.019
KYN	209.12 → 192.17	8	18	0.019
NE	152.12 → 135.10	15	4	0.025
TRP	205.12 → 188.07	8	14	0.019
5-HIAA	192.15 → 146.07	20	42	0.019
5-HT	177.19 → 160.04	10	20	0.019
DA-d4	158.10 → 141.06	10	16	0.025
KA-d5	195.10 → 149.07	20	6	0.019
NE-d6	158.10 → 111.13	18	14	0.025
2H5-L-Trp	210.10 → 192.30	20	10	0.019
5-HT-d4	181.14 → 164.12	8	10	0.019

DA, Dopamine; EPI, Epinephrine; HVA, Homovanillic acid; KA, Kynurenic acid; KYN, Kynurenine; NE, Norepinephrine; TRP, Tryptophan; 5-HIAA, 5-Hydroxyindoleacetic acid; 5-HT, Serotonin.

For the sample preparation procedure, an Ultra-Turrax® T 25 basic (Janke & Kunkel, IKA-Werk, Staufen, Germany) was used for homogenization. Centrifugation was conducted using an Eppendorf 5430 centrifuge, and evaporation was achieved with a Centrifugal Vacuum Evaporator (SALM AND KIPP, RVC 2–33 CO plus). Filtration was performed using an Ostro 96-well plate (25 mg 1/pg), a 2 mL Square Collection Plate (Waters™), and a Positive Pressure-96 Processor. In short, the wet weighed brain tissue (entire right hemispheric prefrontal cortex or hippocampus) was transferred into a 15 mL centrifugal tube and 2 mL of MilliQ water was added. Next, the sample was homogenized using the Ultra-Turrax for 10 s with a 30-s rest, repeated three times. The steel homogenization rod was cleaned before each new sample using chloroform followed by ethanol and water. Of the homogenized brain mixture, 150 μL was transferred and mixed with 15 μL of internal standard (IS) solution (40 ng/mL) into a 1.5 mL Eppendorf tube. Afterwards, 110 μL of the homogenized mixture was pipetted onto an Ostro plate (Waters™), and 300 μL of acetonitrile solvent was added. The Ostro plate, with a Square collection plate (2 mL) beneath it, was placed on a positive pressure apparatus (60 bar, 1 min) to push the liquid through. From each well, 100 μL was transferred to a novel Eppendorf tube and put in the centrifugal evaporator at 45 °C to achieve complete dryness. Finally, 200 μL of 0.6 % FA was added for resuspension, ready for injection. This procedure was performed in duplicate. The ratios between KYN/Trp, 5-HT/Trp, 5-HIAA/5-HT, and HVA/DA in the hippocampus and prefrontal cortex were used as a measure of catabolic turnover.

### 3.8 Statistical analysis

Statistical analyses were performed using R Studio 4.2.2 (R Core, Team, 2022) and SAS 9.4 (SAS Institute Inc., Cary, NC, USA). Gambolling and scamper, hopping, pivoting, rolling, and tossing head were summed into one variable called individual play behaviour, and pushing, nudging, and running together were aggregated into social locomotory behaviour. Tail biting, ear biting, and other damaging behaviours were aggregated into damaging behaviours, and fighting and attack were aggregated into aggressive behaviour. Behavioural frequencies were calculated as frequency per pig per hour, and behavioural durations were calculated as percentage of the total observation time.

Behavioural frequencies were analysed with a generalized linear mixed effects model (GLIMMIX in SAS) with a Poisson distribution and Log link function, and a multiplicative overdispersion parameter. Behavioural durations were analysed with a generalized linear mixed effects model (GLIMMIX in SAS), with a binomial distribution and logit link function, and a multiplicative overdispersion parameter. Both models included sanitary conditions, dietary choice, interaction sanitary conditions × dietary choice, and batch as fixed effects.

Salivary cortisol of individual piglets was analysed in a linear mixed model (with ‘lmer’ function from the R package ‘lme4’) including a fixed effect of sanitary conditions, dietary choice, day (d 2, 11, 18), their interactions, and batch, and a random effect of pen.

Blood parameters and brain neurotransmitters of individual piglets were analysed in a linear mixed model (with ‘lmer’ function from the R package ‘lme4’) including a fixed effect of sanitary conditions, dietary choice, interaction sanitary conditions × dietary choice, batch, and a random effect of pen.

To examine the relationship between plasma Trp and brain Trp and 5-HT concentrations a Pearson's correlation analysis was performed (CORR in SAS) on log-transformed data, and the significance of the correlation was evaluated with a two-tailed test at an alpha level of 0.05. To examine these relationships corrected for the effects of the treatments, correlation analysis was performed on the residuals of the variables, extracted with a general linear model (GLM in SAS) with sanitary conditions and choice as fixed effects.

Residual normality and variance homogeneity were evaluated using Shapiro-Wilk tests and visual inspection of the data. As residuals did not meet normality assumptions, data of salivary cortisol, blood parameters, and brain neurotransmitters were log-transformed. *P*-values below 0.05 were considered statistically significant, and P-values between 0.05 and 0.10 as tendencies. In case of significant fixed effects, pairwise comparisons were done using differences between least square means with a Tukey's HSD correction. Data are presented as means ± SD unless stated otherwise.

## 4 Results

On d 8 of batch one, one piglet allocated to the HSC-Dietary choice treatment died from an intestinal torsion and its pen mate was also removed from the experiment to avoid social isolation. Therefore, data from these two piglets were included only until d 8 of the experiment. All other animals remained clinically healthy during the entire experiment.

### 4.1 Behavioural observations

**Fig. 1:**
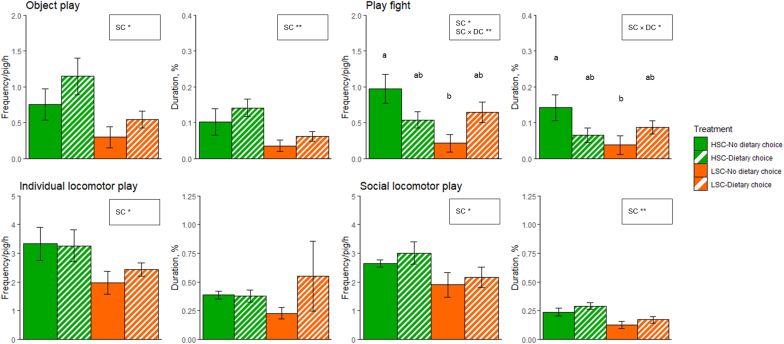
Play behaviour frequency and duration of piglets kept under high (HSC) or low (LSC) sanitary conditions and given either or not a dietary choice between an AA-deficient and an AA-enriched diet on experimental day 16. Only significant effects of sanitary condition (SC), Dietary choice (DC) and/or their interaction (SC × DC) are reported: †P < 0.1, ∗P < 0.05, ∗∗P < 0.01. ^a,b^ In case of a significant interaction effect, means lacking a common superscript letter differ. Values are expressed as mean ± SEM.

Effects of sanitary conditions and dietary choice on play behaviours on d 16 are presented in Fig. 1. Frequency and duration of object play were higher for HSC than LSC piglets (*P* < 0.05 and *P* < 0.01, respectively). Frequency and duration of play fight behaviour were higher in HSC piglets with no dietary choice than in LSC piglets with no dietary choice, with HSC and LSC piglets with a dietary choice in between (sanitary conditions × dietary choice: *P* < 0.01 for frequency and *P* < 0.05 for duration). Individual locomotor play frequency was higher for HSC than LSC piglets (*P* < 0.05), while duration was not affected by sanitary conditions, dietary choice, or their interaction (*P* > 0.05). Social locomotor play frequency and duration were higher for HSC than LSC piglets (*P* < 0.05 and *P* < 0.01, respectively).

**Fig. 2:**
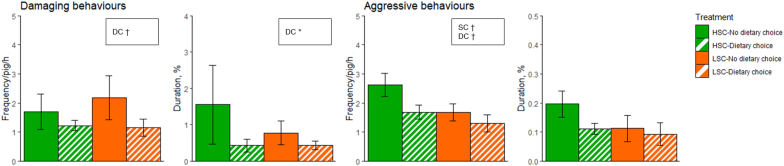
Damaging and aggressive behaviour frequency and duration of piglets kept under high (HSC) or low (LSC) sanitary conditions and given either or not a dietary choice between an AA-deficient and an-AA enriched diet on experimental day 16. Only significant effects of sanitary condition (SC), Dietary choice (DC) and/or their interaction (SC × DC) are reported: †P < 0.1, ∗P < 0.05. Values are expressed as mean ± SEM.

Effects of sanitary conditions and dietary choice on damaging and aggressive behaviours on d 16 are presented in Fig. 2. Damaging behaviour frequency and duration tended to be or were higher for piglets with no dietary choice (*P* < 0.1 and *P* < 0.05, respectively). Frequency of aggressive behaviours tended to be higher for HSC than LSC piglets, and for piglets with no dietary choice compared to piglets with dietary choice (*P* < 0.10 for both sanitary conditions and dietary choice). Duration of aggressive behaviours was not affected by sanitary conditions, dietary choice, or their interaction (*P* > 0.05 for all).

### 4.2 Salivary cortisol

Cortisol concentration in saliva is presented in Table 3. Salivary cortisol was higher on d 4 than on d 11 and d 18 (*P* < 0.001), and was not affected by sanitary conditions, dietary choice, or interactions (*P* > 0.05 for all).

**Table 3:** Salivary cortisol concentration of piglets kept under high (HSC) or low (LSC) sanitary conditions and given either or not a dietary choice (DC) between an AA-deficient and an AA-enriched diet.

	HSC		LSC		*P-values*						
	No dietary choice	Dietary choice	No dietary choice	Dietary choice	SC	DC	SC × DC	Day	SC × Day	DC × Day	SC × DC × Day
Cortisol, μg/dL					0.87	0.37	0.22	**< 0.001**	0.84	0.67	0.31
d 4	9.6 ± 6.7	8.4 ± 6.5	5.3 ± 3.0	14.4 ± 8.9							
d 11	3.0 ± 1.7	4.0 ± 2.6	3.2 ± 1.7	3.3 ± 1.5							
d 18	2.8 ± 2.5	2.6 ± 2.9	2.5 ± 1.5	2.4 ± 1.1							

Abbreviations: HSC, High sanitary conditions; LSC, Low sanitary conditions; SC, Sanitary conditions; DC, Dietary choice. Data are presented as mean ± SD.

### 4.3 Plasma tryptophan, platelet and whole blood serotonin

Plasma Trp, platelet and whole blood serotonin concentration are shown in Table 4. Trp concentration in plasma was higher in HSC than in LSC piglets (*P* < 0.001), and higher for piglets with dietary choice compared to piglets without dietary choice (*P* < 0.05). Platelet 5-HT was higher in HSC than in LSC pigs (*P* < 0.05). Whole blood 5-HT was not affected by sanitary conditions, dietary choice, or their interactions (*P* > 0.05 for all).

**Table 4:** Trp (plasma), serotonin (platelet; whole blood) and monoaminergic metabolite to neurotransmitters ratios (hippocampus and prefrontal cortex) of piglets kept under high (HSC) or low (LSC) sanitary conditions and given either or not a dietary choice (DC) between an AA-deficient and an AA-enriched diet on experimental day 19.

	HSC		LSC		*P-values*		
	No dietary choice	Dietary choice	No dietary choice	Dietary choice	SC	DC	SC × DC
Plasma Trp, nmol/mL	47.1 ± 10.8	53.5 ± 13.6	29.5 ± 11.8	39.3 ± 7.8	**< 0.001**	**< 0.05**	0.27
Platelet 5-HT, nmol/platelet 10^9^	45.2 ± 13.9	42.2 ± 22.5	32.9 ± 12.1	38.3 ± 24.9	**< 0.05**	0.78	0.99
Whole blood 5-HT, nmol/mL	14.3 ± 6.2	11.4 ± 6.4	9.7 ± 5.7	10.3 ± 5.8	0.13	0.93	0.28

Hippocampus, %							
KA/KYN	11.1^ab^ ± 11.7	11.1^ab^ ± 9.6	34.1^a^ ± 32.7	8.5^b^ ± 4.9	0.10	0.12	**< 0.05**
KYN/Trp	3.3 ± 1.0	3.0 ± 1.0	2.1 ± 0.7	2.5 ± 0.9	**< 0.001**	0.66	0.17
KA/Trp	0.4^ab^ ± 0.4	0.3^ab^ ± 0.2	0.7^a^ ± 0.9	0.2^b^ ± 0.1	0.55	0.12	**< 0.05**
5-HT/Trp	3.0 ± 1.3	3.2 ± 1.3	3.2 ± 1.6	3.0 ± 2.0	0.60	0.92	0.38
5-HIAA/5-HT	108 ± 27	91 ± 43	107 ± 33	117 ± 123	0.73	0.31	0.65
HVA/DA	238 ± 166	128 ± 110	176 ± 170	101 ± 87	0.37	**< 0.05**	0.90

Prefrontal cortex, %							
KA/KYN	1.4 ± 1.5	1.7 ± 2.5	1.9 ± 2.6	1.4 ± 0.9	0.71	0.88	0.67
KYN/Trp	7.5^a^ ± 3.8	5.3^ab^ ± 1.2	4.1^b^ ± 1.1	4.7^b^ ± 1.4	**< 0.01**	0.52	**< 0.05**
KA/Trp	0.1 ± 0.1	0.1 ± 0.1	0.1 ± 0.1	0 ± 0.0	0.28	0.90	0.62
5-HT/Trp	1.9 ± 0.8	2.3 ± 1.0	1.8 ± 0.5	2.3 ± 1.5	0.82	0.38	0.92
5-HIAA/5-HT	49 ± 29	42 ± 21	38 ± 15	45 ± 29	0.69	0.90	0.80
HVA/DA	28 ± 32	21 ± 12	36 ± 80	22 ± 21	0.69	0.76	0.98

Abbreviations: HSC, High sanitary conditions; LSC, Low sanitary conditions; SC, Sanitary conditions; DC, Dietary choice; Trp, Tryptophan; 5-HT, Serotonin; KA, Kynurenic acid; KYN, Kynurenine; 5-HIAA, 5-Hydroxyindoleacetic acid; DA, Dopamine; HVA, Homovanillic acid. ^a,b^ Values within a row with different superscripts differ significantly at *P* < 0.05. Data are presented as mean ± SD.

### 4.4 Brain neurotransmitters

**Fig. 3:**
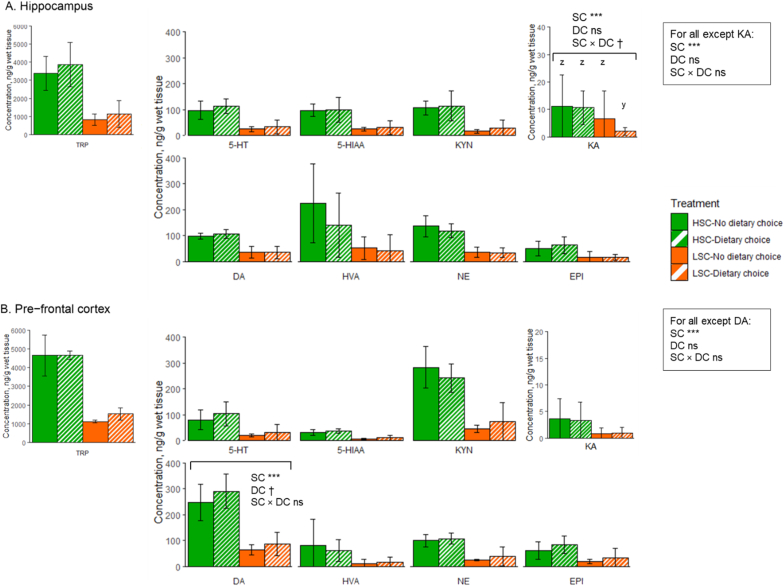
Concentrations of neurotransmitters and their metabolites (ng/g wet tissue) in hippocampus and prefrontal cortex of piglets kept under high (HSC) or low (LSC) sanitary conditions and given either or not a dietary choice between an AA-deficient and an AA-enriched diet on experimental day 19. SC, Sanitary conditions; DC, Dietary choice; Trp, Tryptophan; KYN, Kynurenine; KA, Kynurenic acid; 5-HT, Serotonin; 5-HIAA, 5-Hydroxyindoleacetic acid; DA, Dopamine; EPI, Epinephrine; NE, Norepinephrine; HVA, Homovanillic acid. Only significant effects of SC, DC and/or SC × DC interaction are reported: †P < 0.1, ∗∗∗P < 0.001. ^y,z^ In case of a tendency for an interaction effect, means lacking a common superscript letter differ. Values are expressed as mean ± SD.

Effects of sanitary conditions and dietary choice on neurotransmitters and metabolites in the hippocampus and prefrontal cortex are depicted in Fig. 3. The concentration of Trp, KYN, 5-HT, 5-HIAA, DA, HVA, NE, EPI in the hippocampus was higher for HSC than for LSC piglets (*P* < 0.001 for all). The concentration of KA in the hippocampus was affected by sanitary conditions, with higher levels for HSC piglets, and tended to be affected by the sanitary conditions × dietary choice interaction (*P* < 0.001 and *P* < 0.1, respectively), showing a trend for a lower KA concentration for pigs with a dietary choice than for pigs with no dietary choice, but only under LSC (*P* < 0.1). The concentration of Trp, KYN, KA, 5-HT, 5-HIAA, HVA, NE, EPI in the prefrontal cortex was higher for HSC than for LSC piglets (*P* < 0.001 for all). The concentration of DA in the prefrontal cortex was higher for HSC compared to LSC piglets (*P* < 0.05), and tended to be higher for piglets with dietary choice compared to piglets without dietary choice (*P* < 0.1).

The KA/KYN, KYN/Trp, KA/Trp, 5-HT/Trp, 5-HIAA/5-HT, and HVA/DA catabolic turnover ratios in the hippocampus and prefrontal cortex are presented in Table 4. In the hippocampus, KA/KYN and KA/Trp turnover ratios were affected by the sanitary condition × dietary choice interaction (*P* < 0.05 for both). Piglets without a dietary choice showed a higher turnover ratios compared to piglets with a dietary choice only under LSC, with ratios of piglets under HSC being in between regardless of the dietary choice (*P* < 0.05). In the hippocampus, KYN/Trp was higher for piglets under HSC compared to LSC (*P* < 0.001), and not affected by dietary choice or the sanitary condition × dietary choice interaction (*P* > 0.05 for both). The 5-HT/Trp and 5-HIAA/5-HT turnovers were not affected by sanitary conditions, dietary choice, or their interaction in the hippocampus (*P* > 0.05 for all). HVA/DA was lower for piglets with dietary choice compared to the ones without dietary choice (*P* > 0.05), while there was no effect of sanitary conditions or interaction (*P* > 0.05 for both). In the prefrontal cortex, the KYN/Trp turnover ratio was affected by sanitary conditions and the sanitary condition × dietary choice interaction (*P* < 0.01 and *P* < 0.05, respectively). Piglets without a dietary choice under HSC showed a higher KYN/Trp turnover ratio compared to LSC piglets, regardless of dietary choice, with ratios of piglets with a dietary choice under HSC being in between (*P* < 0.05). The KA/KYN, KA/Trp, 5-HT/Trp, 5-HIAA/5-HT, and HVA/DA turnover ratios in the prefrontal cortex were not affected by sanitary conditions, dietary choice, or their interaction (*P* > 0.05 for all).

### 4.5 Correlations between plasma Trp and brain Trp and serotonin

**Fig. 4:**
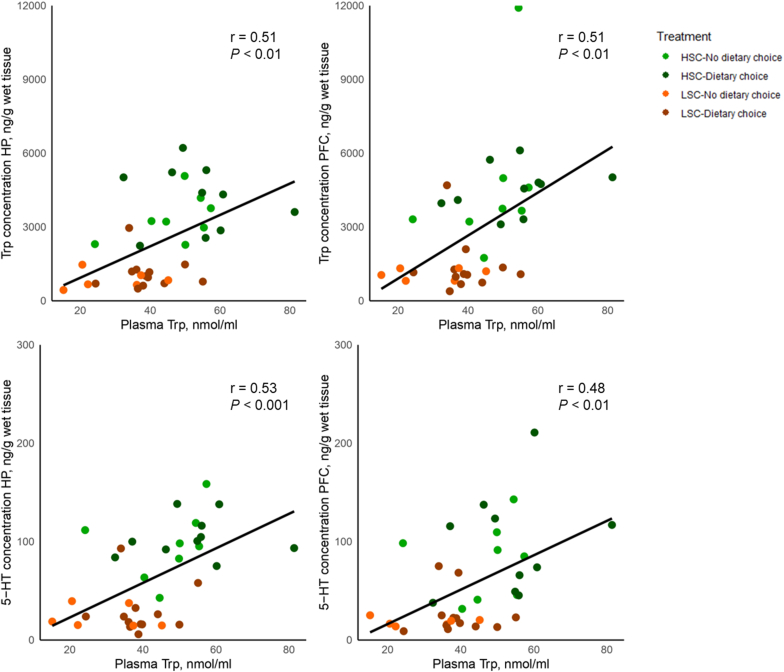
Pearson correlation between Trp in plasma and Trp and serotonin in the brain (hippocampus and prefrontal cortex) of piglets kept under high (HSC) or low (LSC) sanitary conditions and given a choice or not between an AA-deficient and an AA-enriched diet on experimental day 19. HP, Hippocampus; PFC, Prefrontal cortex; HSC, High sanitary conditions; LSC, Low sanitary conditions; Trp, Tryptophan; 5-HT, Serotonin.

The relationship between plasma Trp and brain Trp and serotonin is shown in Fig. 4. Trp concentration in plasma was positively correlated to Trp concentration in the hippocampus and prefrontal cortex (r = 0.51, *P* < 0.01 for both). Trp concentration in plasma was positively correlated with 5-HT concentration in the hippocampus and prefrontal cortex (r = 0.53, *P* < 0.001, and r = 0.48, *P* < 0.01, respectively). When removing the effect of the treatments, however, Trp concentration in plasma did not correlate with Trp concentration in the hippocampus and prefrontal cortex (r = 0.14, *P* = 0.40, and r = 0.11, *P* = 0.50, respectively), nor with 5-HT concentration in the hippocampus and prefrontal cortex (r = 0.01, *P* = 0.96, and r = 0.09, *P* = 0.59, respectively).

## 5 Discussion

The aim of the present study was to investigate the effects of housing piglets under different sanitary conditions and offering either a diet deficient in indispensable AA, or the choice to select between this deficient diet or an AA enriched diet, on behaviour and brain monoamine as well as blood and brain tryptophan (Trp) levels. Low sanitary conditions (LSC) decreased plasma Trp, and concentrations of Trp, monoamines, and their metabolites in the hippocampus and prefrontal cortex. In addition, play behaviour was shown less by piglets kept under LSC compared to piglets under high sanitary conditions (HSC). Providing piglets with a choice between an AA-deficient and an AA-enriched diet increased the blood Trp concentration, decreased the homovanillic acid (HVA)/dopamine (DA) ratio in the hippocampus, and tended to increase hippocampal DA. Also, pigs with this dietary choice tended to show less damaging and aggressive behaviours.

The LSC induced mild inflammation and immune stimulation, reflected in increased numbers of leukocytes, monocytes, neutrophil and eosinophil granulocytes and blood haptoglobin concentrations compared to HSC, as we reported elsewhere (Minussi et al., 2025). This was accompanied by a decrease in feed intake and body weight gain of 31 and 44 %, respectively (Minussi et al., 2025). In the current study, however, no effect of sanitary conditions on salivary cortisol was found in these pigs, despite clear signs of a chronically activated immune system. Possibly, the single time point salivary sampling was insufficient to detect potential differences in basal cortisol levels, whereas the heightened levels on the first sampling day reflected the acute stress of weaning and transport.

The current study demonstrated lower plasma Trp and platelet serotonin (5-HT) concentrations in piglets kept under LSC compared to HSC, and the same trend, albeit not significant, was observed for whole blood 5-HT. Piglets kept under LSC also had lower concentrations of Trp, kynurenine (KYN), kynurenic acid (KA), and 5-HT, DA and their metabolites, and lower norepinephrine (NE) and epinephrine (EPI) in both hippocampus and prefrontal cortex compared to piglets under HSC. Trp concentrations in plasma and brain were positively correlated, similar to another study (Veit et al., 2021), but not when corrected for treatments, suggesting that the raw correlation was mainly caused by the treatment effects. The same held for the correlation between plasma Trp and brain 5-HT.

The 32 % lower Trp concentration in blood observed in piglets raised under LSC aligns closely with the 31 % reduction in feed intake (Minussi et al., 2025), suggesting that the lower Trp levels may be a result of a reduced overall intake of Trp. Trp in the blood is normally tightly regulated and Trp depletions can be corrected by degrading body proteins (Riedel et al., 2002). However, this compensatory mechanism might have been insufficient under LSC, as the additional Trp made available by degradation of body proteins might have been preferentially utilized to support immunological process in piglets kept under LSC (Le Floc'h et al., 2004).

The reduced neurotransmitter levels in the brain of piglets under LSC align with studies in which acute sickness was experimentally induced by intraperitoneal or intravenous injection with lipopolysaccharide (LPS). Mice and pigs exposed to such a sickness challenge were found to have lower Trp, 5-HT, KYN, NE, and DA concentrations in different brain areas (frontal cortex, hypothalamus, hippocampus) compared to non-challenged animals (Zhu et al., 2015; Nordgreen et al., 2018; Veit et al., 2021). The amplitude of the effects of sanitary conditions in the current study is greater than what was observed in the aforementioned studies, which can be the result of the type of challenge applied. LPS injection is an acute and temporary challenge compared to the one imposed by housing piglets under LSC, which results in a more chronic reduction in health status and feed intake. Remarkably, whereas LSC produced a decrease in blood Trp of around 30 %, the effects on brain Trp, monoamines and their metabolites were much higher (approximately a three-fold reduction). This might either be due to a higher peripheral Trp catabolism, particularly through the inflammation-induced activation of the indoleamine 2,3 deoxygenase 1 (IDO) pathway or a less efficient uptake of Trp in the brain (Nordgreen et al., 2018; Veit et al., 2021).

Catabolic turnover ratios of most neurotransmitters were not affected by sanitary conditions, indicating overall lower levels of both the parent neurotransmitter and its metabolite in pigs kept under LSC, without affecting the regular enzymatic conversion rate. The KYN/Trp turnover ratios were, however, higher in the hippocampus and prefrontal cortex of pigs kept under HSC, albeit in the prefrontal cortex this only held for the pigs without the dietary choice. This increased conversion of Trp to KYN was unexpected because inflammation generally increases the Trp to KYN conversion in the brain (Le Floc'h and Seve, 2007), however, this might be due to a higher demand of nicotinamide adenine dinucleotide (NAD^+^), the end metabolite of the kynurenine pathway which is needed for energy production in the cells (Moffett and Namboodiri, 2003), in piglets under HSC with higher activity levels and higher growth.

Sickness influences behaviour in different ways: it reduces general activity, decreases the motivation to engage in (social) activities, and may lead to a more negative affective state (Munsterhjelm et al., 2019). Piglets kept under LSC had a lower frequency and duration of object play and individual and social locomotory play compared to piglets kept under HSC. Low sanitary conditions also reduced play fighting, albeit only in pigs without diet choice. Rats showed reduced play behaviour after an LPS challenge (Gaykema et al., 2008), although the impact of poor sanitary conditions leading to subclinical inflammation on play behaviour specifically has to the best of our knowledge hardly been studied. Play behaviour has been associated with a positive affective state (Reimert et al., 2013), and is not or hardly displayed by animals when they are sick or stressed (see Held and Špinka (2011) for review). The reduction in play behaviour in pigs kept under LSC could thus reflect a shift towards negative affective states. The effects of sanitary conditions on behaviour may partly be mediated through their impact on the availability and metabolism of Trp, ultimately affecting levels of 5-HT in the brain (Le Floćh and Seve, 2007). Brain 5-HT is important in regulating mood and emotion, and low 5-HT neurotransmission is generally associated with more negative affective states (Hensler, 2010). Additionally, the low levels of play could be associated with the general reduction of activity that accompanies the sickness response. This low activity is an adaptive response to retain energy for metabolically costly responses of the immune system, such as fever (Hart, 1988; Johnson, 2002), but it has been observed that also mild LPS-induced inflammation without clinical signs decreases activity of pigs (Johnson and Von Borell, 1994; Krsnik et al., 1999; Nordgreen et al., 2018; Miller et al., 2019; Munsterhjelm et al., 2019; Veit et al., 2021). The presumed lower activity level in pigs kept under LSC might also explain why these pigs tended to show a lower frequency of aggressive behaviours, which was in contrast with expectations based on another study (van der Meer et al., 2017) in which no effects of sanitary conditions on aggression were found. It was observed, however, that aggression frequently stemmed from antagonism for access to the toy available in the pen. As pigs under HSC played more with the objects, this might be another explanation for the tendency of more frequent aggressive behaviours in these pigs. In addition, as in the present experiment a chain with a plastic toy attached was the only play material available, the resources for playing were limited, which could have exacerbated aggressive behaviours (Andersen et al., 2000; Verdon and Rault, 2024). No effects on other damaging behaviours were found, contrary to other studies reporting an increase in tail and/or ear biting after or during an immune challenge, whether via LPS injection (Munsterhjelm et al., 2019; Veit et al., 2021) or by imposing LSC (van der Meer et al., 2017). Given that the low sanitary conditions reduced plasma Trp and platelet 5-HT, the absence of an effect on damaging behaviours is also in contrast with studies reporting a negative impact of low dietary Trp levels on these behaviours in pigs (Li et al., 2006; Martínez-Trejo et al., 2009; Poletto et al., 2010, 2014), or low platelet 5-HT levels in tail biting pigs (Ursinus et al., 2014). In contrast to this study, however, in these previous studies the reduced levels of Trp and/or 5-HT were not caused by immune stimulation.

Piglets with a dietary choice had higher plasma Trp levels compared to piglets without dietary choice, while no effect was found for platelet and whole blood 5-HT. Dietary treatment also did not affect brain neurotransmitter concentrations, except for a higher HVA/DA ratio, related to a trend for a higher DA level in the prefrontal cortex of pigs provided with a choice compared to the ones given the AA-deficient diet only. There were a few interactions with sanitary conditions, i.e. the KYN/Trp ratio in the prefrontal cortex was not affected by sanitary conditions in pigs with a dietary choice, while it was higher for high than for low sanitary status in the pigs with no dietary choice. Also, pigs with a dietary choice tended to have lower KA concentrations in the hippocampus, but under LSC only. The effects of dietary choice on brain neurotransmitters and behaviour were experimentally linked to the ability of piglets to choose between the AA-deficient and AA-enriched diet. It has previously been demonstrated that pigs can detect a dietary deficiency in single (Ettle and Roth, 2004, 2005, 2009; Roth et al., 2006; Suarez et al., 2012) or multiple (Minussi et al., 2024) AA and adjust their feed selection accordingly. Given the higher usage of AA under LSC for immune processes, dietary requirements for AA are assumed higher than those under HSC. In support of this, a previous study reported that AA supplementation counteracted the negative effect of a low sanitary status on ear biting (van der Meer et al., 2017). It was therefore expected that pigs exposed to LSC with a dietary choice in the present study would have a higher intake of the AA-enriched diet than pigs kept under HSC to fulfil their increased requirements (van der Meer et al., 2017). However, piglets consumed only low amounts of the AA-enriched diet compared to the AA-deficient one in both sanitary conditions, and, as a consequence, AA intake did not significantly differ between the choice and no-choice piglets (Minussi et al., 2025), albeit an effect on plasma Trp was demonstrated in the current study. In addition, the preference for the AA-enriched diet did not differ between pigs under HSC and LSC over the whole experimental period, although the relative intake of the AA-enriched diet increased with time under HSC, while it decreased under LSC (Minussi et al., 2025). This resulted in a numerically higher AA intake for piglets with a dietary choice under high and lower under low sanitary conditions compared to piglets without dietary choice in the final days of the experiment (Minussi et al., 2025). Possibly, the mild sickness state of the pigs kept under LSC, apart from reducing their feeding motivation, shifted their preference towards a high-carbohydrate, low protein diet due to 5-HT-mediated effects on mood (see Leigh Gibson (2006) for review). Given the low intake of the AA-enriched diet, the relatively small effects of dietary choice on brain neurotransmitters and behaviour are, therefore, not surprising.

While sanitary conditions mainly affected play behaviour, having a dietary choice reduced time spent on damaging behaviours and tended to reduce the frequency of these behaviours, as well as the frequency of aggression. It has been shown that supplementation of dietary Trp above requirements for growth can reduce stress response and aggressive behaviours in pigs (Koopmans et al., 2005; Li et al., 2006; Martínez-Trejo et al., 2009; Poletto et al., 2014), and the dietary choice group had slightly increased blood Trp levels. Dopamine is also associated with behaviour: some studies have shown that reduced DA concentration in the brain can be linked to learning and memory impairment in rats (Sato et al., 2020), and changes in HVA/DA turnover have been associated with damaging behaviour such as tail biting in different areas than the prefrontal cortex of pigs (Valros et al., 2015), and feather pecking in laying hens (van Hierden et al., 2002). Given the low intake of the AA-enriched diet, however, the reduction of damaging and aggressive behaviours in the dietary choice piglets might also be due to other reasons. The presence of two different diets in the pen could have stimulated the piglet's olfaction, serving as a sensory enrichment and partly fulfilling motivation to explore (Schild and Rørvang, 2023). Providing a diverse feeding regime has been proven to have positive effects on exploratory behaviour in piglets (Middelkoop et al., 2019). As damaging behaviours are forms of exploratory behaviour redirected towards pen mates in the absence of adequate sensory stimulation (Fraser, 1987; Jensen et al., 1993; Moinard et al., 2003), providing pigs with opportunities for exploration might be inherently rewarding and fulfilling their behavioural needs. Dopamine in the prefrontal cortex has been shown to play a role in the processing of sensory stimuli, exploration and motivation (Belujon and Grace, 2015), but it is unknown whether the exposure to different sensory stimuli in pigs provided with a dietary choice relates to the higher DA levels and lower HVA/DA turnover in their prefrontal cortex.

A limitation of the current study is that only female piglets were used. It is worth noting that sex-specific differences in behaviour, even at a young age (Melotti et al., 2011), and immune and stress responses to pathogenic challenges (Williams et al., 2009) were found in pigs. In addition, sex effects on brain neurotransmitter levels have been reported in other species (de Vries, 1990). As the present study focuses solely on female piglets, the results should therefore be interpreted within this context, and further research is needed to elucidate whether they also hold for male pigs.

In conclusion, low sanitary conditions reduced play behaviour in pigs, possibly reflecting a more negative affective state, as well as blood Trp, platelet 5-HT and monoaminergic neurotransmitter concentrations in the hippocampus and prefrontal cortex. Pigs with the possibility to select between an AA-deficient and an AA-enriched diet had a lower HVA/DA turnover in the hippocampus and tended to show less damaging and aggressive behaviours. This indicates that a decline in health status negatively influences Trp and brain neurotransmitter concentrations and behaviour of pigs, while providing pigs with the option for self-supplementation of AA has only a limited impact on these response parameters.

## CRediT authorship contribution statement

**Ilaria Minussi:** Writing – review & editing, Writing – original draft, Visualization, Supervision, Methodology, Investigation, Formal analysis, Conceptualization. **Walter J.J. Gerrits:** Writing – review & editing, Supervision, Methodology, Funding acquisition, Conceptualization. **Alfons J.M. Jansman:** Writing – review & editing, Supervision, Methodology, Funding acquisition, Conceptualization. **George Troupakis:** Writing – review & editing, Methodology, Investigation, Formal analysis. **Marlies Diepeveen-de Bruin:** Writing – review & editing, Methodology, Formal analysis. **Yannick Vermeiren:** Writing – review & editing, Supervision, Methodology, Funding acquisition. **J. Elizabeth Bolhuis:** Writing – review & editing, Supervision, Methodology, Funding acquisition, Conceptualization.

## Funding

This study is part of the Public Private Partnership LWV19175 “Improving low-protein diets to prevent behavioural problems in pigs” and funded by the Dutch Ministry of Agriculture, Nature and Food, De Heus, ForFarmers, and Eurolysine. Gerrit Grijns Initiative (GGI) seeding grant is also acknowledged. The funders had no role in data collection and analysis, decision to publish, or preparation of the manuscript.

## Declaration of competing interest

All authors declare no conflicts of interest.

## Data Availability

Data will be made available on request.
