# Supplementary material for: Impact of sanitary conditions and dietary amino acids on behaviour and brain monoamine levels in piglets
Source: Brain Behav Immun Health. 2025 Aug 5;48:101076. doi: 10.1016/j.bbih.2025.101076 (PMC12356033; doi:10.1016/j.bbih.2025.101076)
Supplement: Multimedia component 1 [file mmc1.docx]

Supplementary material

Table S1

Ct values of faecal samples collected from four commercial farms in different compartments of weaned piglets and used to create the low sanitary conditions. Samples in bold were excluded from the pooled faeces.

|  | *C. perfringens* toxin CPA | *C. perfringens* toxin CPB | *C. perfringens* toxin CPB2 | *E. coli* virulence factor F4 | *E. coli* virulence factor F41 | *E. coli* virulence factor F5 | *E. coli* virulence factor F6 | Rota virus A |
| --- | --- | --- | --- | --- | --- | --- | --- | --- |
| Farm 1 |  |  |  |  |  |  |  |  |
| Sample 1 | 32.2 | nd | nd | nd | nd | nd | nd | 20.9 |
| **Sample 2** | **32.5** | nd | **35.1** | nd | nd | nd | nd | **20.6** |
| Sample 3 | nd | nd | nd | nd | nd | nd | nd | 25.5 |
| Farm 2 |  |  |  |  |  |  |  |  |
| Sample 1 | nd | nd | nd | nd | nd | 32.7 | nd | 23.0 |
| Sample 2 | nd | nd | nd | nd | nd | nd | nd | 27.3 |
| Sample 3 | nd | nd | nd | 34.4 | nd | 34.6 | nd | 26.5 |
| **Sample 4** | nd | nd | nd | **30.5** | nd | **29.6** | nd | 22.3 |
| Farm 3 |  |  |  |  |  |  |  |  |
| **Sample 1** | 34.6 | nd | nd | **27.4** | nd | nd | nd | **19.4** |
| Sample 2 | nd | nd | nd | nd | nd | nd | nd | 23.5 |
| Sample 3 | nd | nd | nd | nd | nd | nd | nd | 26.5 |
| Farm 4 |  |  |  |  |  |  |  |  |
| **Sample 1** | 31.9 | nd | nd | **24.7** | nd | nd | nd | **15.5** |
| Sample 2 | nd | nd | nd | nd | nd | nd | nd | 24.4 |
| Sample 3 | nd | nd | nd | nd | nd | nd | nd | 21.9 |

Nd = non detectable. For E. coli, Ct < 30 appears to be clinically relevant. For Rota virus A, Ct < 20 appears to be clinically relevant.

Table S2

Ingredient composition and analysed nutrient composition of the experimental diets.

|  | Diets | |
| --- | --- | --- |
|  | LP^-1^ | LP^+2^ |
| Ingredients (g/kg as fed) |  |  |
| Barley | 300.0 | 300.0 |
| Wheat | 279.7 | 279.7 |
| Maize | 200.0 | 200.0 |
| Soybean meal | 68.0 | 68.0 |
| Wheat middlings | 25.0 | 25.0 |
| Cane molasses | 20.0 | 20.0 |
| Maize starch | 50.0 | 25.1 |
| Soybean oil | 10.0 | 11.5 |
| Limestone fine | 10.0 | 10.0 |
| Monocalcium phosphate | 8.6 | 8.6 |
| Sodium propionate | 5.0 | 5.0 |
| Potato protein | 5.0 | 5.0 |
| Sodium chloride | 3.0 | 3.0 |
| Vitamin + mineral mix^3^ | 2.0 | 2.0 |
| Potassium | 2.4 | 2.4 |
| Axtra PHY 20000 FTU/g | 0.1 | 0.1 |
| Sodium bicarbonate | 0.001 | 0.001 |
| L-Lysine HCl | 5.6 | 11.2 |
| L-Threonine | 1.9 | 4.4 |
| DL-Methionine | 1.9 | 4.4 |
| L-Threonine | 1.9 | 4.4 |
| L-Valine | 0.9 | 4.1 |
| L-Leucine | - | 4.4 |
| L-Isoleucine | 0.3 | 2.6 |
| L-Tryptophan | 0.6 | 1.6 |
| L-Histidine | 0.2 | 1.6 |
|  |  |  |
| Nutrient composition (g/kg) |  |  |
| Dry matter^4^ | 881 | 884 |
| NE_2015_^5^ (MJ/kg) | 10.4 | 10.4 |
| Analysed crude protein^6^ | 123 | 142 |
| Analysed starch^7^ | 493 | 469 |
| SID amino acids (total analysed)^8^ | |  |
| Lys | 8.8 (9.3) | 13.2 (13.6) |
| Met+Cys | 5.7 (5.4) | 8.1 (8.0) |
| Thr | 5.7 (5.7) | 8.6 (8.5) |
| Trp | 1.9 (2.0) | 2.9 (2.9) |
| Val | 6.2 (6.1) | 9.2 (12.2) |
| Ile | 4.7 (4.5) | 7.0 (8.8) |
| Arg | 6.1 (6.2) | 6.1 (6.3) |
| Phe | 5.5 (5.4) | 5.5 (5.5) |
| His | 2.8 (2.8) | 4.2 (3.8) |
| Leu | 8.9 (8.7) | 13.2 (13.1) |
| Tyr | 3.6 (3.5) | 3.6 (3.6) |

Abbreviations: LP = Low protein; DM = Dry matter; CP = Crude protein; SID = Standardized ileal digestible.

^1^ LP^-^: A low protein diet formulated to be deficient in Lys, Met, Thr, Trp, Val, Leu, Ile, His by 20% compared to requirements for maximal body weight gain (CVB, 2020).

^2^ LP^+^: A low protein diet formulated to be with Lys, Met, Thr, Trp, Val, Leu, Ile, His at +20% above requirements for maximal body weight gain (CVB, 2020).

3 Vitamin + mineral mix composition (/kg diet): vitamin A (10000 IU), vitamin D_3_ (2000 IU), vitamin E (40 mg), vitamin K_3_ (1.5 mg), vitamin B_1_ (1.0 mg), vitamin B_2_ (4.0 mg), vitamin B_6_ (1.5 mg), vitamin B_12_ (20 µg), niacin (30 mg), D-pantothenic acid (15 mg), choline chloride (150 mg), folic acid (0.4 mg), biotin (0.05 mg), iron (100 mg), copper (20 mg), manganese (30 mg), zinc (70 mg), iodate (0.7 mg), selenium (0.25 mg).

4 Analysed with ISO 6496 (ISO, 1999).

5 NE = net energy, MJ/kg (CVB, 2022).

6 Analyzed with ISO 16634-1 (ISO, 2005a).

7 Analyzed with ISO 15914 (ISO, 2004).

^8^ SID AA based on table values for feed ingredients for pigs (CVB, 2020), in brackets the total AA concentration analysed with NF EN ISO13903 (ISO, 2009) and ISO13904 (ISO, 2005b).

***Feed analyses***

Diets were analysed for DM according to ISO 6496 (ISO, 1999), starch according to ISO 15914 (ISO, 2004), and CP according to ISO 16634-1 (ISO, 2008). The AA composition of the diets was analysed with a JLC-500/V AminoTac Amino Acid Analyzer (Jeol, Croissy-sur-Seine, France) according to NF EN ISO 13903 (ISO, 2009). For cysteine and methionine determination, the samples were oxidized with performic acid prior to hydrolysis. The AA were separated by ion exchange chromatography and determined by reaction with ninhydrin. Tryptophan was determined by reversed-phase C18 high performance liquid chromatography (HPLC) according to NF EN ISO13904, MOD.0094 (ISO, 2005b). These chemical analyses were executed in duplicate, and when the coefficient of variation was >5%, analyses were repeated.

Table S3

Optimisation for measurement of Trp with mass spectrometry.

| Component | Cone voltage | Collision energy | Parent ion | Daughter ion | Dwell time |
| --- | --- | --- | --- | --- | --- |
|  | (V) | (eV) | (m/z) | (m/z) | (ms) |
| Trp | 18 | 12 | 205 | 188 | 50 |
| Trp d5 | 18 | 12 | 210 | 193 | 50 |
|  |  |  |  |  |  |

**References**

Central Bureau for Livestock Feeding (CVB), Nutrient requirements and feed ingredient composition for pigs. Lelystad, The Netherlands, 2020.

Central Bureau for Livestock Feeding (CVB), Chemical composition and nutritional values of feedstuffs. Lelystad, The Netherlands, 2022.

ISO, 1999. 6496: Animal feeding stuffs: determination of moisture and other volatile matter content. In International Organization for Standardization, Geneva, Switzerland.

ISO, 2004. 15914: Animal feeding stuffs: enzymatic determination of total starch content. In International Organization for Standardization, Geneva, Switzerland.

ISO, 2005a. 5983-1: Animal feeding stuffs: determination of Nitrogen content and calculation of crude protein content—Part 1: Kjeldahl method. In International Organization for Standardization, Geneva, Switzerland.

ISO, 2005b. 13904: Animal feeding stuffs: determination of Tryptophan content. In International Organization for Standardization: Geneva, Switzerland.

ISO, 2008. 16634-1: Determination of the total nitrogen content by combustion according to the Dumas principle and calculation of the crude protein content — Part 1: Oilseeds and animal feeding stuffs. In International Organization for Standardization, Geneva, Switzerland.

ISO, 2009. 13903: Animal feeding stuffs: determination of amino acids content. In International Organization for Standardization, Geneva, Switzerland.
